# Consumption and exchange in Early Modern Cambodia: NAA of brown-glaze stoneware from Longvek, 15th–17th centuries

**DOI:** 10.1371/journal.pone.0216895

**Published:** 2019-05-13

**Authors:** Martin Polkinghorne, Catherine Amy Morton, Amy Roberts, Rachel S. Popelka-Filcoff, Yuni Sato, Voeun Vuthy, Pariwat Thammapreechakorn, Attila Stopic, Peter Grave, Don Hein, Leng Vitou

**Affiliations:** 1 Archaeology, Flinders University, Adelaide, South Australia, Australia; 2 College of Science and Engineering, Flinders University, Adelaide, South Australia, Australia; 3 Department of Planning and Coordination, The Nara National Research Institute for Cultural Properties, Nara, Nara Prefecture, Japan; 4 Department of Archaeology and Prehistory, Ministry of Culture and Fine Arts, Phnom Penh, Phnom Penh, Cambodia; 5 Arthur M. Sackler Gallery and the Freer Gallery of Art, Washington, D.C., United States of America; 6 Reactor Irradiations, Radioisotopes and Radiotracers, Australian Nuclear Science and Technology Organisation, Lucas Heights, New South Wales, Australia; 7 Archaeology and Palaeoanthropology, University of New England, Armidale, New South Wales, Australia; Universita degli Studi di Milano, ITALY

## Abstract

An evaluation of the geochemical characteristics of 102 storage jar sherds by *k*_*0*_-neutron activation analysis (*k*_*0*_-NAA) from archaeological contexts in Cambodia and reference samples from stoneware production centres in Thailand provides a new perspective on regional and global trade in mainland Southeast Asia. Identification of seven geochemical groups enables distinctions between production centres, and articulation of their role in trade between northern and central Thailand, South China and Cambodia. Storage jars from Thailand and South China are known in archaeological contexts worldwide because of their durability and intrinsic functional and cultural values. Evidenced by a novel application of *k*_*0*_-NAA, analogous stoneware sherds at Longvek connect the Cambodian capital to a global trading network. Additional proof of ceramics from an undocumented Cambodian kiln demonstrates the gradual and complex transition between the Angkorian past and the Early Modern period.

## Introduction

In the sunset of pre-modern agrarian kingdoms, Southeast Asia was undergoing great changes that formed the nation-states we know today. Traits that defined Angkor (c.900 C.E.– c.1450 C.E.)—large scale but low-density urbanism, monumental stone architecture, landscape-scale infrastructure, and purported centralised economic systems—were no longer transforming the physical world of Cambodia at an equivalent scale. The Early Modern period, between the late 15th and late 18th centuries, was a decisive epoch in Southeast Asia [1–7]. For the first time there were direct trade routes between Europe and Asia. Southeast Asia was *the* hub of international trade between East and West. While the demise of Angkor from the 14th century is the subject of on-going study, a long-held belief is that a new focus on maritime trading opportunities in the Early Modern period resulted in people moving away from the inland capital to pursue new coastal economic opportunities related to an expansion in international trade [6, 8].

However, for this region of mainland Southeast Asia, the transition between the Angkorian and Early Modern periods remains poorly defined. Our understanding of economic activity, in particular trade, is hindered by the unreliability of historical evidence including indigenous sources and regional trading records, and the paucity of studied material culture proxies. The prominence of the magnificent built remains at Angkor, coupled with the rich corpus of inscriptions, has provided fertile grounds for archaeological and historical research. However, this focus has done little to advance wider perspectives on the dynamics of the era after Angkor, for example the impact of the Early Modern period transition on local economic, political, or social trajectories. A new emphasis on this period could fill a significant gap in our current knowledge for both the region, the "post-Angkorian" capitals, and the Early Modern period. At Longvek we have material evidence of continuity and development in global commerce embodied in trade ceramics from China, Japan, Thailand, and Vietnam [9, 10]. While consumers in the Angkorian period used ceramics produced in Angkorian-controlled territories, following the decline of Angkor and its associated craft industries, including high-fired ceramic production, utilitarian stoneware was imported from regional and international neighbours. The material record in Cambodia can offer important evidence on the Ming export ban [11], and the coincident escalation in Thai and Vietnamese ceramic production [12–14].

While there are alternative techniques of quantitative multi-elemental analysis, the application of Neutron Activation Analysis (NAA) to archaeological artefacts, specifically ceramics, has an emerging utility in Asian archaeology [15–20] and complements archaeological studies of this period in Cambodian history [21–25]. We present the results of elemental characterisation (*k*_*0*_-NAA) of sherds of brown-glaze stoneware storage jars excavated from Longvek, the 16th and 17th century capital of Cambodia, and reference samples from production complexes in northern and central Thailand. Interpretation of the compositional analysis reveals an assemblage of stoneware storage jars from Thailand, South China, and Cambodia and new information on consumption and exchange in Early Modern Cambodia.

## Background

### Early modern Southeast Asia

In Southeast Asia, the Early Modern period is usually defined as an expansive era for the development of international commerce, coastal regional centres, and new forms of technologies and craft industries. Predominantly based on historical sources, transformations of resource use, religious systems, and state structures were first associated with colonial perspectives of European exploration and mercantilism. Post-colonial interpretations situate local agency as a fundamental mechanism of change [1–7, 26, 27]. Nevertheless, the character of these developments remains uncertain. Similarly, Stark [28] has noted a paucity of archaeological research on this period. Archaeological studies on stoneware ceramics from production sites and the ceramic cargoes of shipwrecks are the exception to this lacuna. By specifying the chronology, quantity, and technology of ceramics researchers have made advances in considering the interplay between production, political economy, and trade in the Early Modern period [11, 29–31, 12–14, 32–34].

### Longvek

**Fig 1:**
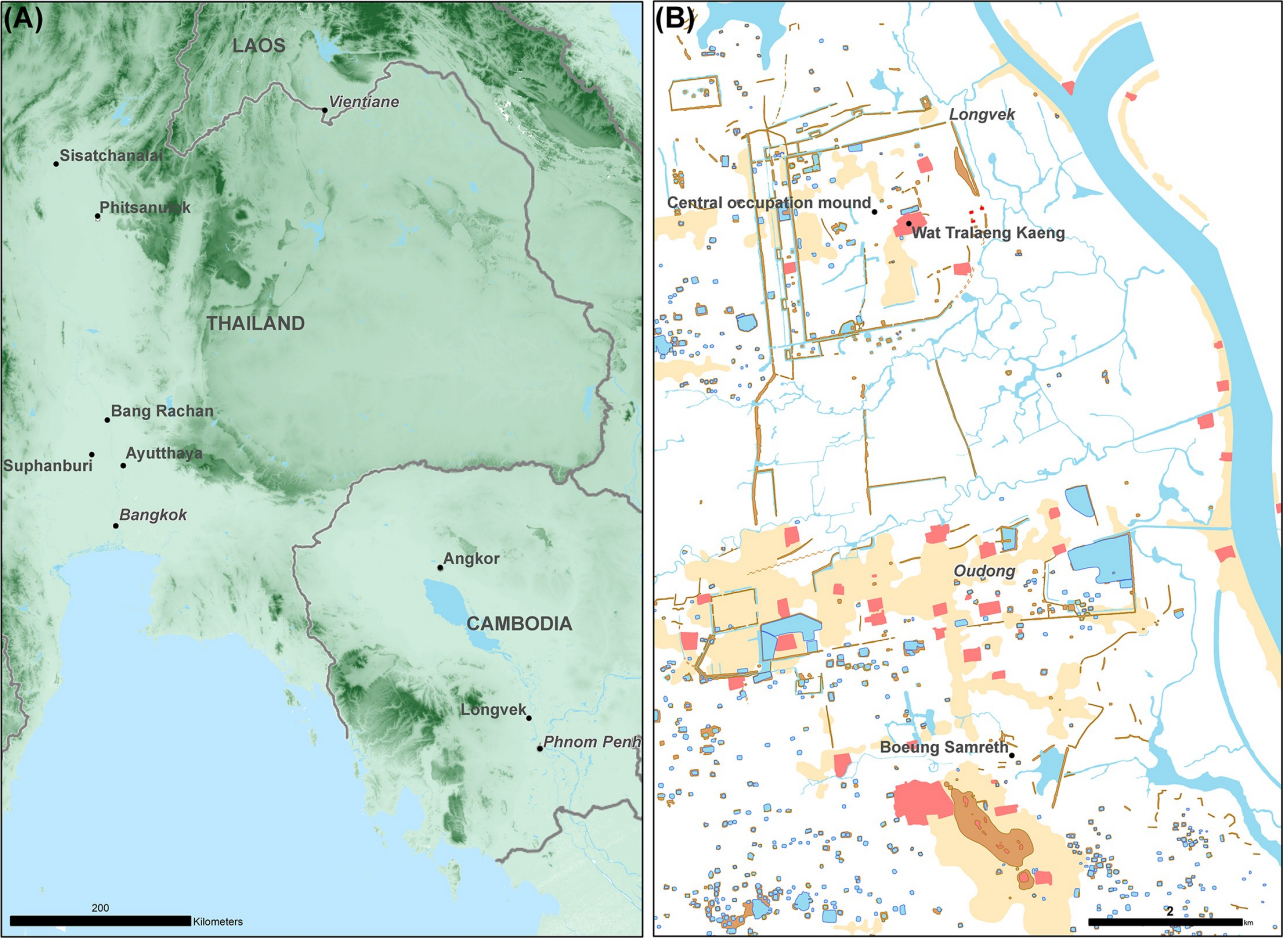
Maps showing locations of prodution and consumption of brown-glaze stoneware storage jars. (A) Production sites in northern and central Thailand (Sisatchanalai, Bang Rachan, Suphanburi, Phitsanulok) and consumption site in Cambodia (Longvek). This map includes Shuttle Radar Topography Mission (SRTM) data which has been released and distributed without restrictions. (B) Preliminary archaeological map of Longvek / Oudong showing locations of storage jar samples—Wat Traleang Kaeng, Central occupation mound, Boeung Samreth. Courtesy Cambodian Archaeological LiDAR Initiative.

Longvek was Cambodia's most important political, religious, and commercial centre throughout the 16th and 17th centuries [8, 35–37]. The settlement was reportedly founded by King Ang Chan I (reigned 1516/17 C.E. or 1526 C.E.– 1566 C.E.) who returned from Ayutthaya, where he had been residing during a civil war, to claim Cambodian royal authority at a new capital about 40 kilometres upriver from Phnom Penh [8, 38]. The site is characterised by series of earthen embankments that form a seven-square-kilometre rectilinear citadel against a natural barrier of flooded paddy and the Tonle Sap River to the east (Fig 1). Significantly, Longvek is the largest settlement of a centralised Cambodian state after Angkor.

Our principal understandings of Longvek are from interpretations of the Royal Cambodian Chronicles [38–42]. These texts are problematic as historical documents because they are largely written from oral histories long after the events they allege to report. While the Chronicles declare that the major buildings of Longvek were constructed around 1530, epigraphy and material culture demonstrate occupation in the area at least from the pre-Angkorian period (c.700 C.E.) [43, 44]. Preliminary interpretation of archaeological survey and excavation dates new and major phases of occupation beginning from the 15th century [45–47].

Longvek, like many sites of equivalent periods, has large quantities of porcelain tableware [9, 10]. Highly durable, portable and beautiful, blue and white porcelains and their replicas are among the most recognisable and researched material forms of Early Modern Southeast Asian trade [48–56]. However, research on utilitarian ceramics like storage jars can also provide critical evidence on regional production, exchange, and consumption [31, 57–64]. At Longvek, sherds of stoneware are a common feature of the ceramic assemblage [46,47] and different kinds of brown-glaze stoneware sherds from large storage jars have been recovered from numerous sites.

### Storage jars

Large ceramic jars were used and re-used as the containers of trade and storage across the pre-modern world. In Southeast Asia and China, between the 9th and 18th centuries, stoneware jars produced in a wide range of sizes were principally employed as utilitarian vessels to transport all kinds of materials, including foodstuffs and manufactured objects [64]. Stoneware storage jars survive in the material record because of their durability. However, there are limitations in considering jars as proxies for consumption and exchange. Jars are used for long periods of time, sometimes hundreds of years, and may be unreliable for dating even when linked to robust stratigraphies. Similarly, although the numbers of stoneware sherds may be numerous, because jars are mostly large, and can break into many fragments, the minimum number of individuals remains low.

Inspired by the earliest Chinese examples, the majority of storage jars produced in China and Southeast Asia are characterised by glossy yellowish brown to very dark brown glazes, and correspondingly sherds are easily recognisable in archaeological contexts. While visual inspection of Longvek sherds have led to a hypothesis that they are of Thai, South Chinese, and Cambodian origin, elemental analysis is the only reliable method to accurately discriminate geochemical groups and infer production provenience.

### Compositional analyses

The association between the elemental composition of an artefact and the original material source was outlined by Weigand, Harbottle and Sayre [65] as the ‘Provenience Postulate’. To establish the veracity of provenience, the difference in chemical composition between separate sources must exceed, in some recognisable way, the differences observed within a given source [65]. Following this concept, NAA of ceramics has successfully investigated compositional groupings by comparing ceramics from an unknown provenience with a reference collection of ceramics from a known provenience. Techniques such as Portable X-Ray Fluorescence (pXRF) are increasingly available to practitioners, however this method is susceptible to surface effects such as roughness, and particle size—parameters that are difficult to standardise for archaeological samples. NAA was selected because it offers high precision, accuracy, sensitivity, and the ability to analyse small samples. By comparison to pXRF and XRF, NAA is matrix independent, has parts-per-million (ppm) / parts-per-trillion (ppt) limits of detection, especially for the rare earth elements (REE), which are critical for distinguishing groups in geological materials and in this study [66–70].

Grave has led numerous geochemical compositional studies on Early Modern Southeast Asian and Chinese stoneware [31, 33, 34, 60, 70]. Using the analytical techniques of PIXE-PIGME and ICP-OES on shipwreck ceramics, Grave considered the relationship between variability in production outputs and economic change [31, 33, 60, 70]. When considering Southeast Asian stoneware Grave and Maccheroni’s [31] PIXE-PIGME analysis of samples from Bang Rachan (Maenam Noi) and Sisatchanalai concluded that ceramics were elementally indistinguishable. However, it was unclear if the insignificant geochemical discrimination was a consequence of analytical techniques limitations or the presence of compositionally homogenous sediments [18].

On the subject of Early Modern Chinese ceramics, recent analytical studies have made great advances in characterising the geochemical composition of Chinese porcelain [50, 71–74]. Yet, there has been little interest in storage jars produced in South China at the same time because of their relatively diffuse chronological resolution and apparently mundane appearance [63]. The research described in this paper represents the first application of NAA to Southeast Asian material culture of the Early Modern period and from geochemical characterisation proposes the identification of stoneware storage jars from Thailand, South China, and Cambodia.

## Methods and materials

### Sampling strategy

One hundred and two stoneware sherds (approx. 2–3 g each) of brown-glaze stoneware fragments from the consumption site of Longvek and production complexes at Sisatchanalai, Bang Rachan, Suphanburi and Phitsanulok were sampled for this study (S1 Table). All necessary permits were obtained for the described study, which complied with all relevant regulations. The sample and permission information are listed below. NAA is a destructive method and there are no residual samples requiring deposition.

**Fig 2:**
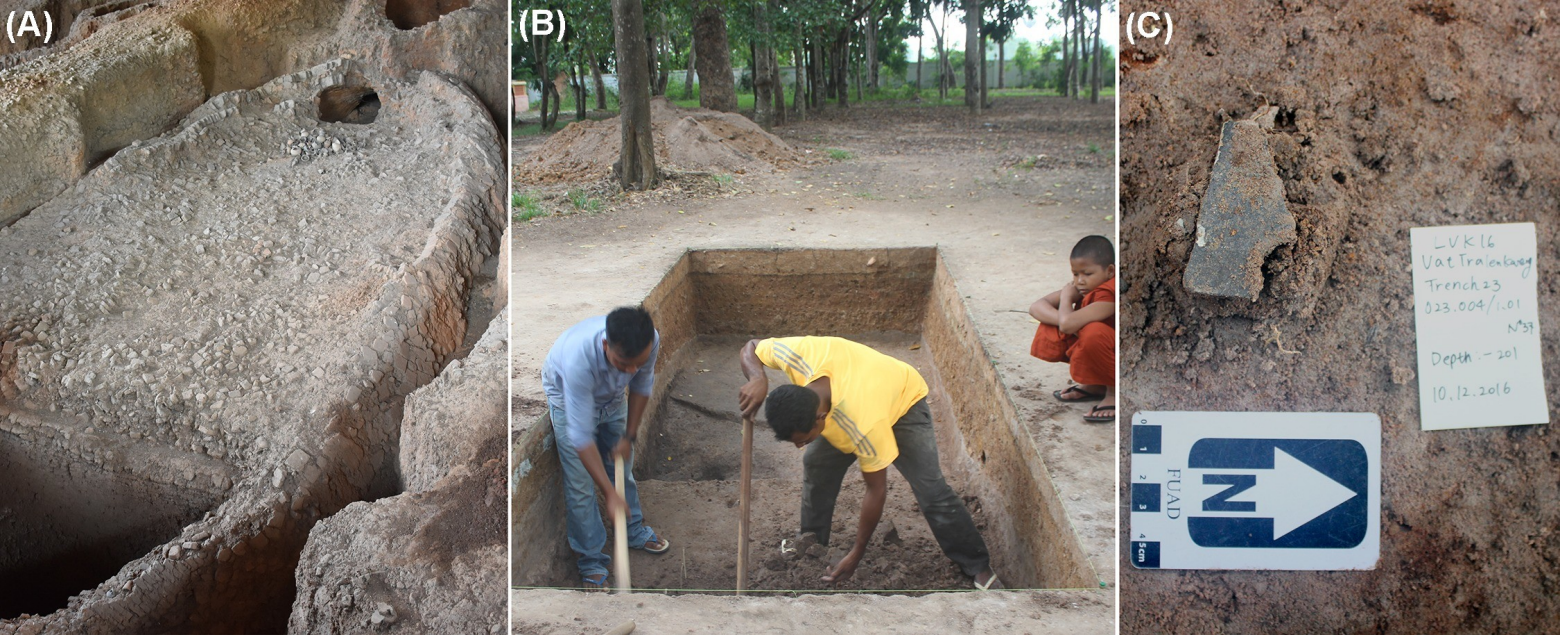
Production and consumption sites of brown-glaze stoneware storage jars. (A) Bang Rachan (Maenam Noi) Kilns no. 1 & 5. Image credit: Martin Polkinghorne. (B) Wat Tralaeng Kaeng—Trench 23. Image credit: Sok Keo Sovannara. (C) Brown-glaze sherd in-situ at Longvek—BCG100, South China. Image credit: Sok Keo Sovannara.

1. Eighty-eight sherds collected from archaeological contexts from Longvek, north of Phnom Penh (15th– 17th centuries) (see Figs 1 and 2). Permmision to excavate and analyse samples was granted to Flinders University (Flinders) and The Nara National Research Institute for Cultural Properties (NABUNKEN) from the Royal Government of Cambodia, Ministry of Culture and Fine Arts (MoCFA).

2. Three sherds from the Bang Rachan production site (see Fig 1, Fig 2) collected by Pariwat Thammapreechakorn (Southeast Asian Ceramics Museum, Bangkok University) with permission from the Fine Arts Department in 1995.

3. Eleven sherds and fragments of stoneware vessels from Sisatchanalai, Suphanburi and Phitsanulok collected by Dr Don Hein (Thai Ceramics Dating Project) in the late 1970s and early 1980s (see Fig 1) [13]. Permision to excavate and analyse samples was granted to the Thai Ceramics Dating Project from the National Research Council of Thailand, and the Fine Arts Department.

### Archaeological contexts

In 2015 and 2016 collaborative archaeological investigations were conducted at Longvek by the Royal Government of Cambodia, Ministry of Culture and Fine Arts, the National Research Institute for Cultural Properties Nara (NRICPN), and Flinders University. Samples were collected from surface surveys and stratigraphic excavations at three sites: 1. Wat Tralaeng Kaeng; 2. A central occupation mound known by its contemporary industrial use as "the brick factory", and; 3. the copper-base alloy production site of Boeung Samreth (Fig 1). The Royal Cambodian Chronicles state that Wat Traleang Kaeng was constructed in the first decades of the 16th century, though earlier epigraphic evidence, sandstone and laterite blocks, and the remains of a moat suggest the site was formerly an Angkorian temple. Wat Tralaeng Kaeng is a complex archaeological site, and has probably borne continuous occupation undergoing numerous modifications. Test pit excavations have recognised a stratigraphic layer with apparently in-situ artefacts from the Early Modern period with corresponding diagnostic Chinese porcelain predominantly dating to the mid-16th century.

The centre of the Longvek citadel is characterised by a raised platform or mound of approximately 2 ha, which presently accommodates a series of three contemporary kilns devoted to manufacturing bricks. Located in the approximate geographical centre of Longvek, the occupation mound is linked by its proximity to Wat Traleang Kaeng, and another small Buddhist terrace to the east. Comparatively large quantities of high-value and unusual trade porcelain, dating mainly between the mid-16th and early 17th centuries, demonstrate this was a site of elite occupation [9, 10]. Similar to Wat Traleang Kaeng, disturbance, reuse, and contemporary occupation are obstacles to understanding the chronological sequence. However, preliminary interpretation of radiocarbon ages date landscape modification here to the 15th century [46, 47].

Boeung Samreth is a copper-base alloy production site situated at the foot of Phnom Preah Reach Top—a small hill which became the necropolis for several of Cambodia's Early Modern kings. Described in the Royal Cambodia Chronicles, this site was discovered as a result of land clearance and shallow ploughing with a mechanical excavator carried out to establish a tourist recreation area [75]. Although the stratigraphic context of the workshop appears to have been largely destroyed, remains of at least one metal working structure is intact, and the site is abundant with technical ceramics (crucibles, moulds, and furnace fragments), casting evidence, slags, and foundry waste. Pedestrian survey across an area of 1 ha collected large quantities of diagnostic Chinese porcelain mostly dating between the mid-16th and 17th centuries, and brown-glaze stoneware presented for this study.

### Reference samples

The Bang Rachan (Maenam Noi) kilns, located in Singburi province, are named after the river on which they are situated. Their primary output are recognised as brown-glaze four-lug storage jars in various shapes and sizes, which have been commonly mistaken for products from Sisatchanalai and associated sites. Production is known from the 15th century until the mid 18th century and the kiln outputs have been recognised at terrestrial and shipwreck sites, across Southeast Asia, and as far away as Japan, Africa, the Middle East, and the South Atlantic Ocean [20, 59, 76–85]. Sisatchanalai is considered to be the largest and have the longest operational life of any ceramic production centre in mainland Southeast Asia. Production activities began in the 12th century with considerable expansion from the 14th and 15th centuries until the 17th century when production appears to terminate [12, 14, 33, 86, 87].

Published data on the geology and soil geochemistry around the production centres of Bang Rachan and Sisatchanalai is fragmentary. The underlying geology of the Bang Rachan region consists of Quaternary fluvial deposits [88]. The Bang Rachan kiln complex lies on a flood plan which may have provided the raw materials needed for ceramic production. Similarly, the underlying geology of the Sisatchanalai region also consists of Quaternary fluvial deposits, however it is in close proximity to the Permian Ratburi geological formations which consist of limestone, dolomitic limestone and chert [88]. While geological classifications are informative, future studies will benefit from detailed geochemical characterisations of the Bang Rachan and Sisatchanalai regions. Attempting to determine all possible clay deposits used for ceramic production is beyond the scope of this study due to the multiple variables involved (e.g. the depletion of a deposit over time), however future studies could sample and analyse local clay deposits to assess the variability and correspondence of locally available clays.

### Sample preparation

**Fig 3:**
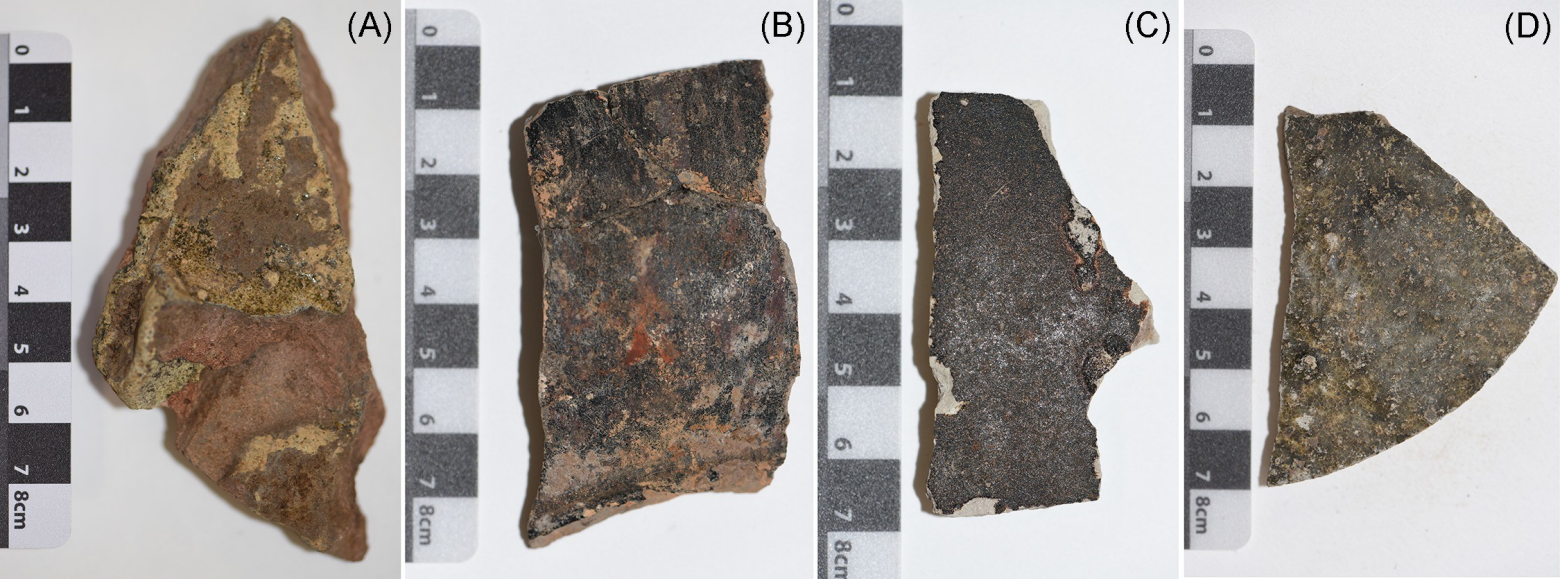
Trade ceramics at Longvek. (A) Sisatchanalai brown-glaze stoneware sherd (BGC028, Central occupation mound—Trench 15). (B) Bang Rachan brown-glaze stoneware sherd (BGC097, Wat Tralaeng Kaeng—Trench 23). (C) South China brown-glaze stoneware sherd (BGC100, Wat Tralaeng Kaeng—Trench 23). (D) Cambodian brown-glaze stoneware sherd (BGC078, Central occupation mound—Trench 21). Image credits: Catherine Morton.

As noted by Glascock, Neff and Vaughn [89] and Grave et al. [18] sample preparation required for NAA of ceramic samples is minimal. The stoneware samples analysed in this study were prepared according to the procedures outlined in previous studies [67, 89]. Prior to sample preparation all artefacts were catalogued and photographed (Fig 3). Fragments of approximately 1.5 x 1.5 cm were removed from larger sherds by either cutting or breaking. In order to remove possible surface contaminants and the glaze from the sample, the surface was removed using a dremel with a tungsten carbide high-speed burr. The burring was conducted within a customised perspex box to contain dust and limit the possibility of contamination. After each sample was burred the perspex box and dremel attachment were washed with water/detergent and dried using ethanol. Samples were washed with deionized water and dried. Each cleaned sample contained within two thick plastic bags was crushed to 10,000 PSI in a hydraulic hand pump (Power Team, SPX USA). Clean plastic discs were also placed between the bags and the piston rod and work plate to distribute the pressure and mitigate splitting of the bags which could lead to contamination of the sample. The piston rod and work plate of the hydraulic hand pump were cleaned with deionized water and ethanol in between each sample crushing as an additional precautionary measure. Where the hydraulic crushing did not produce the desired consistency the sample was further pulverised into a powder using an agate mortar and pestle which was cleaned between each sample. Approximately 1 g of each powdered sample was then placed into labelled vials for submission to the Australian Nuclear Science and Technology Organisation (ANSTO).

### Sample analysis

Sample analysis was performed using established *k*_*0*_-NAA methodology for archaeological ceramic samples using the 20MW OPAL research reactor at ANSTO [67, 90–94]. Samples underwent both short and long residence time experiments to determine a total of over 50 elements per sample (see S1 Data). Three standard reference materials were also measured for inter-laboratory comparison (NIST2711a, NIST688, NIST278). The samples (approximately 50–100 mg) were packed for both short and long irradiations by ANSTO, who also performed the gamma-spectrometry and elemental analysis. The remaining powdered samples were kept for future reference and possible additional analysis.

### Data analysis

Data analysis followed the methods outlined in Popelka-Filcoff et al. [92, 93] and Bland et al. [67]. Compositional comparisons were made using the GAUSS MURRAP routine (MURR Archaeometry Laboratory 2014). This study used principal component analysis (PCA), one of several commonly used pattern recognition techniques applied to archaeological geochemical data [67, 68, 95]. PCA was employed to identify compositional groups and to consider which elements within the dataset were affecting variability. Statistical analyses were carried out on base-10 logarithms of 31 elemental concentrations. Some elements were not used because their concentrations were either incomplete across the dataset or below the limit of detection (e.g. Cl, Re and Gd). The use of log concentrations rather than raw data compensates for differences in magnitude between major elements, such as iron and sodium, and trace elements, such as rare earth elements [67]. Elements that influenced data variability were additionally subjected to agglomerative hierarchical cluster analysis to clarify the way that samples in the same group were more similar than to those in other groups.

## Results

**Fig 4:**
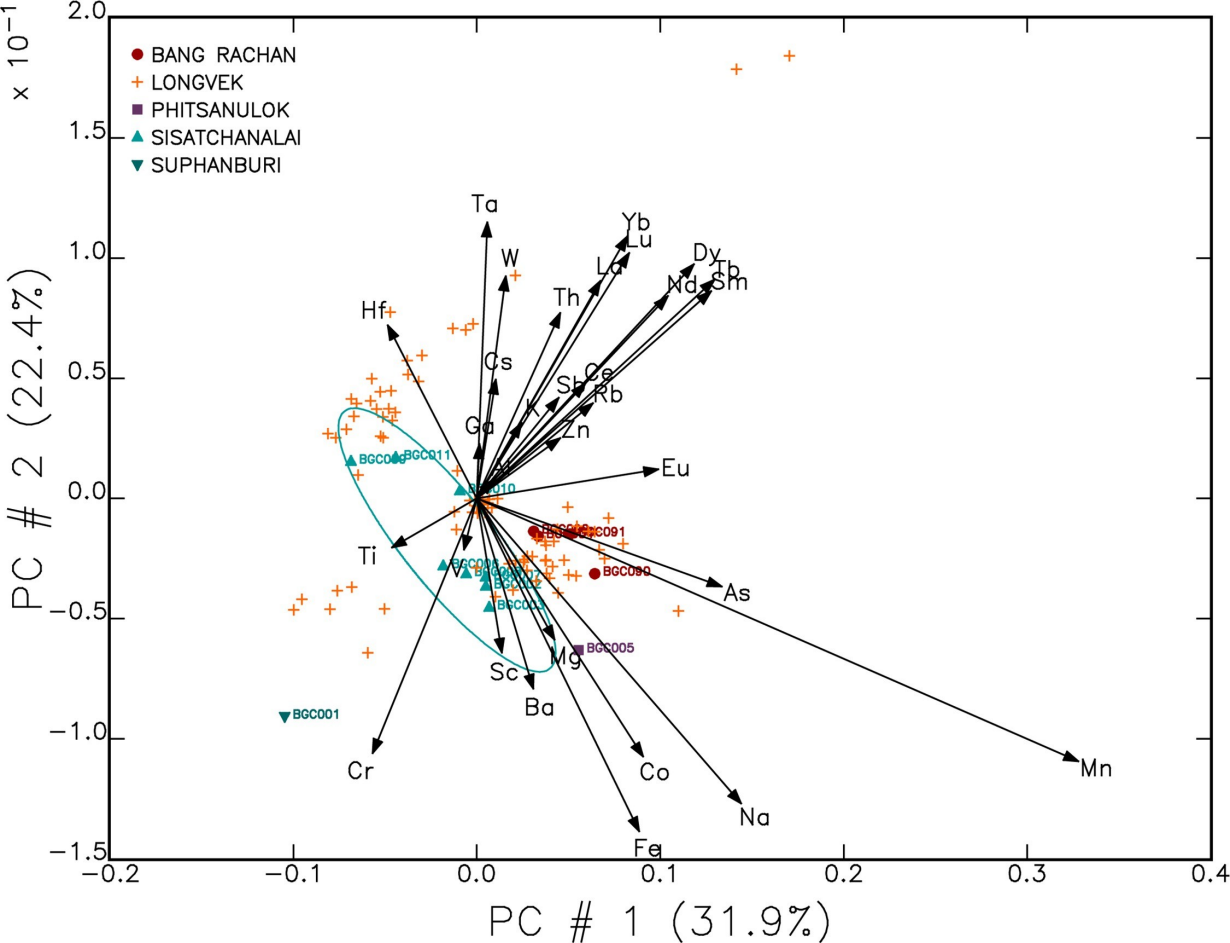
Biplot of first two principal components, describing 54.85% of data set cumulative variation.

The PCA was able to account for 96.86% of the variability within the first 10 PCAs (see S2 Table). The results from the PCA for the ceramic dataset shows high variability for the following elements: Fe, Mn, Na and Co (see Fig 4). Iron is one of the main elements driving variability within the dataset and, along with antimony, was recognised by Grave et al. [19] by stepwise discriminant function analysis as among the elements that best distinguish stoneware kilns in Cambodia. In contrast to other analytical techniques (e.g. pXRF), NAA detects antimony at 0.1 ppm or below in the ceramic matrix, and can be considered as discriminatory element. In the present study antimony is orthogonal to iron in PCA 1 suggesting that these two elements can be employed to analyse the data. Correspondingly, iron and antimony were selected to investigate groupings of the samples based on hierarchical cluster analysis and bivariate plots.

**Fig 5:**
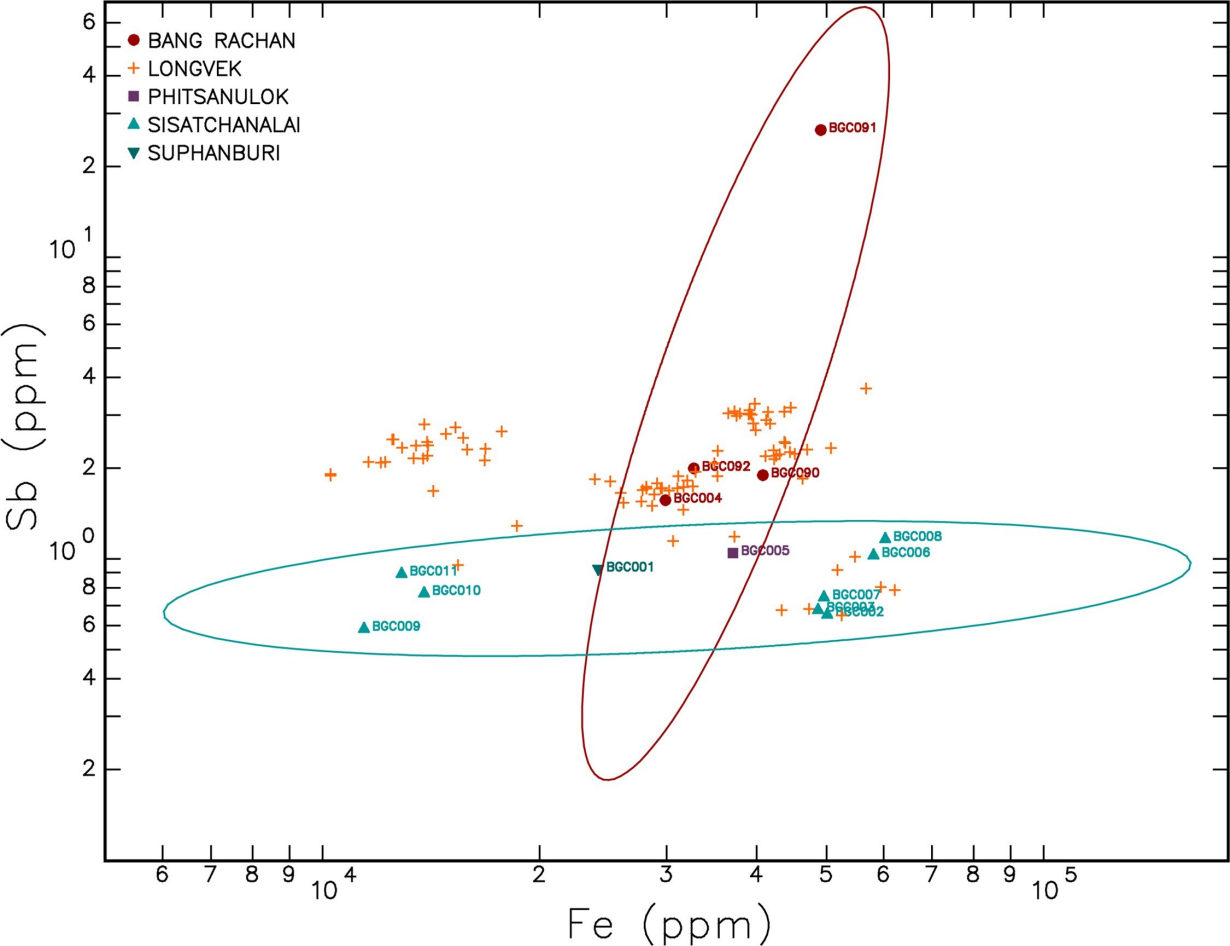
Bivariate plot of Fe and Sb concentrations with emphasis on Bang Rachan and Sisatchanalai.

Following Grave et al. [19] Fig 5 illustrates a bivariate plot of Fe and Sb concentrations for the ceramic dataset. The reference ceramic samples from Bang Rachan and Sisatchanalai are labelled and the remaining samples were from Longvek. Fig 5 illustrates a number of Longvek samples falling outside the Bang Rachan and Sisatchanalai ellipses.

**Fig 6:**
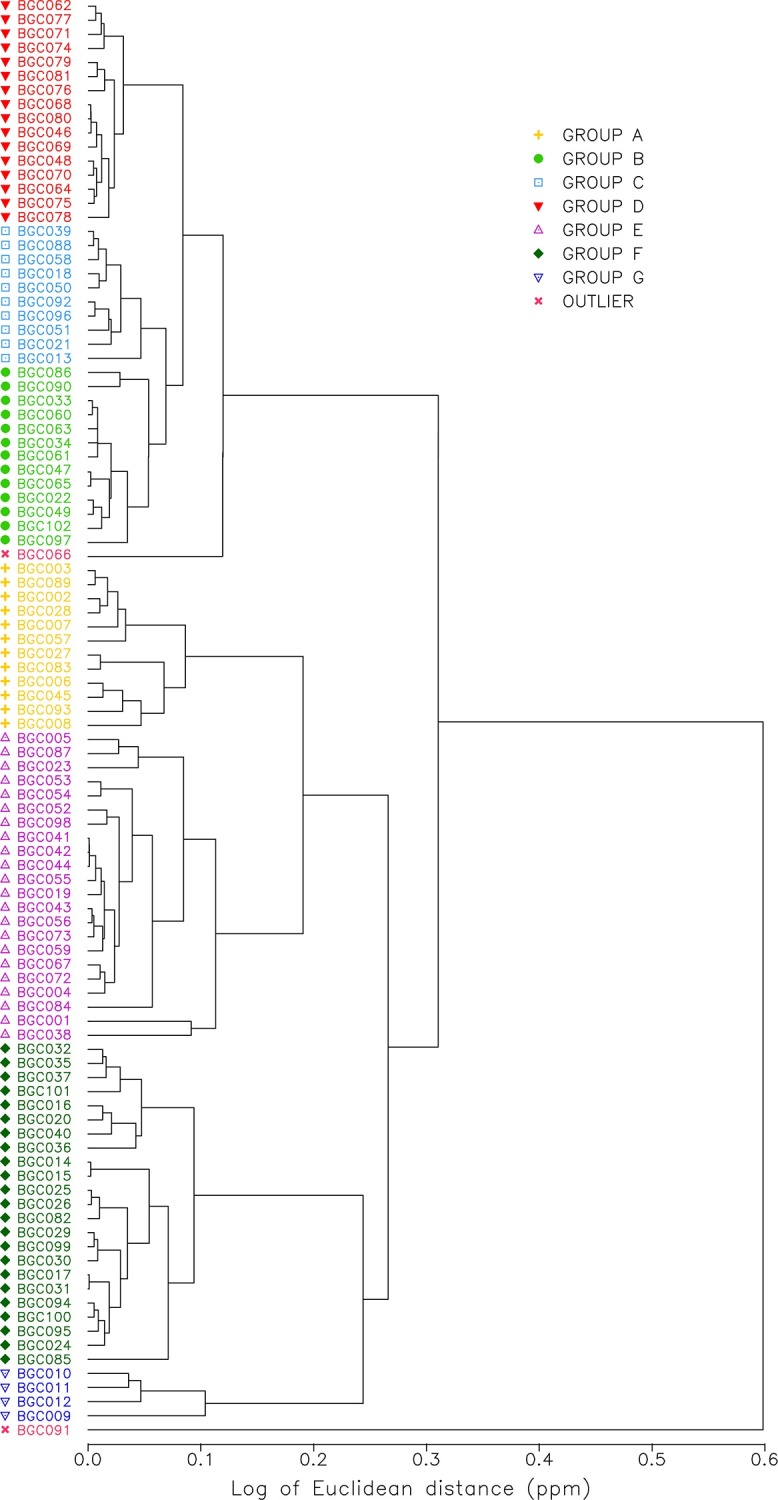
Hierarchical cluster analysis identifying seven groups (A to G).

**Fig 7:**
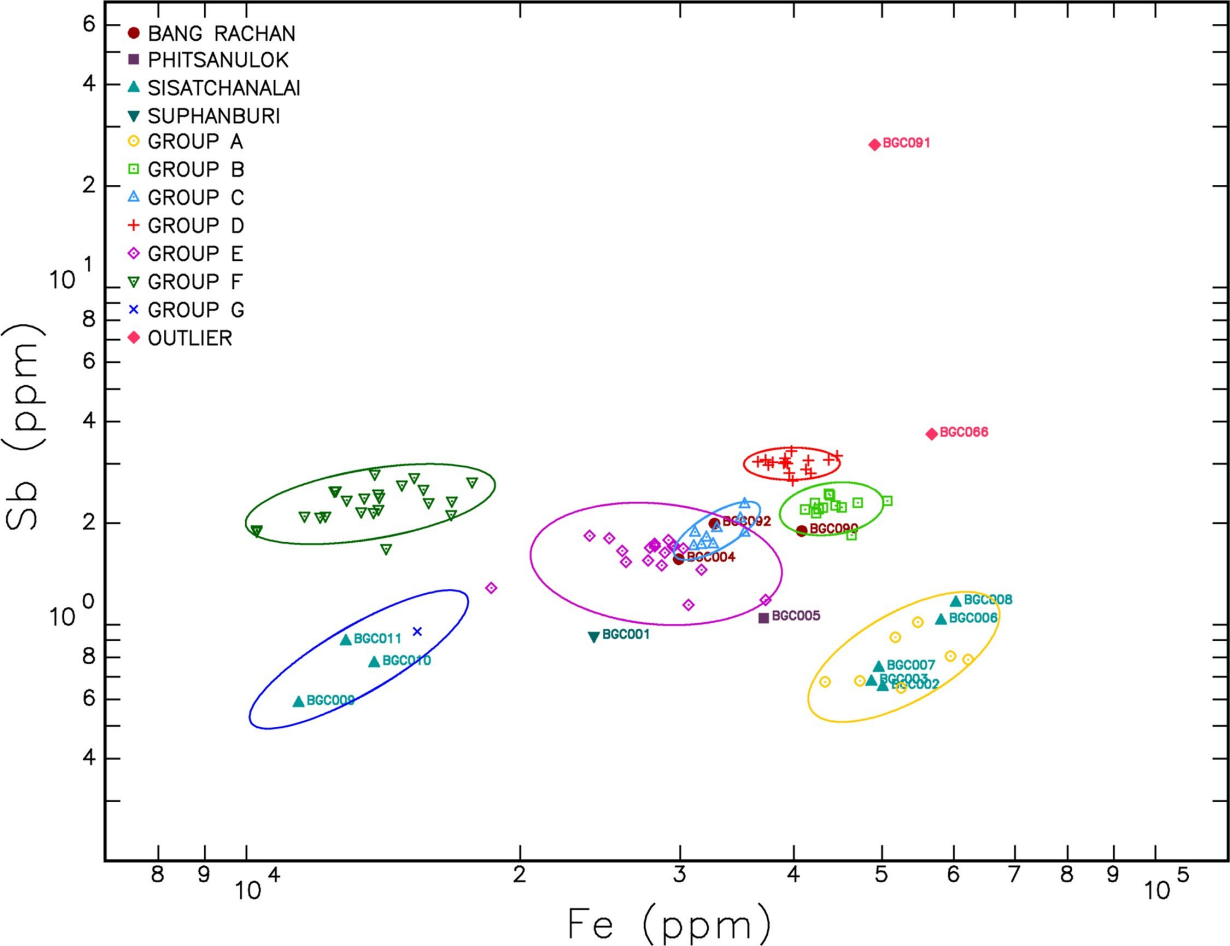
Bivariate log-log plot of antimony and iron concentrations classified by cluster analysis compositional groups.

In order to investigate the clustering of the samples a hierarchical cluster analysis based on iron and antimony was used and recognised seven groups (A through to G) (see Fig 6). A bivariate plot of iron and antimony with the samples grouped into those identified by the hierarchical cluster analysis is presented in Fig 7.

## Discussion

Analysing the composition of 102 storage jar sherds by *k*_*0*_-NAA discriminated seven geochemical groups making it possible to infer manufacture and trade from Thailand, South China, and an undocumented Cambodian kiln (Table 1, and below). Because archaeological contexts of these artefacts can span more than three hundred years it is difficult to reconstruct a high resolution chronological framework. Nevertheless, radiocarbon dating, and association with diagnostic blue and white Chinese porcelain, indicates that the deposition of these fragments occurred between the early-15th and mid-17th centuries [46, 47].

**Table 1:** Summary description of sherds in this study.

	Reference Samples	Wat Tralaeng Kaeng (Longvek)	Central occupation mound (Longvek)	Boeung Samreth (Longvek)	Total
Thailand (Sisatchanalai) *Groups A & G*	8	1	6		16
Thailand (Bang Rachan) *Groups B*, *C & E*	4	4	30	7	44
Thailand (Pitsanoluk) *Group E*	1				1
Thailand (Suphanburi) *Group E*	1				1
South China *Group F*		6	16	1	23
Cambodia (unknown kiln) *Group D*		2	14		16
Outlier			1		1
Total	14	13	67	8	102

### Discriminating Thai kilns (Groups A, B, C, E and G)

NAA of the Longvek and reference samples demonstrates that the Cambodian capital was receiving brown-glaze stoneware storage jars originating from kilns in present-day Thailand. In an advance of former analytical studies of brown-glaze stoneware [31] the results of this research have successfully distinguished between Bang Rachan and Sisatchanalai production centres. Group A and G both contain Sisatchanalai reference samples and are linked to that production centre. Group G has considerably less Cr (55.33 ppm) than the other groups (e.g., Group D 106.93 ppm). However group G has almost twice the La, Sm, Tb, Dy, Yb and Lu than the other groups. The division between the Sisatchanalai reference samples may relate to a difference in raw materials, changes in production over time, or variability in the geochemistry near these kiln production centres.

Groups B, C and E contain Bang Rachan reference samples. Group B and C are compositionally the closest of all the groups (see Fig 6). The ellipses of Group C and E overlap in Fig 7 and demonstrate similar compositions. Each Group, B, C and E contain one of the Bang Rachan reference samples indicating that they are all likely from the Bang Rachan kiln complex area. Discrimination of three groups might indicate geographic or temporal differences in the origin of raw materials. A refined compositional chronology is required to verify this hypothesis.

### Discriminating Chinese brown-glaze (Group F)

From the combination of visual observations that discern yellowish-brown and brown-glazes and a tightly defined geochemical group (Group F) we propose the first known elemental characterisation of storage jars from the provinces of Guangdong or Fujian in South China. It is probable that each production centre has a discrete compositional signature and we are cautious to posit a precise geographic or temporal attribution. Based on the hierarchical cluster analysis (Fig 6) it is clear that Group F is significantly different to Groups A to E, which are more geochemically similar to each other. Group F has significantly less sodium (Na) on average (775.54 ppm) than the other groups (e.g. Group B has 3433.08 ppm).

Large quantities of stoneware storage jars were manufactured in South China from the 9th century onwards for local use and export. Both yellowish-brown and brown-glazed jars dated between the 14th and early 17th centuries are known from dated sites in Guangdong, Hong Kong, and Macau [63]. Notably, several yellowish-brown glazed jars are known from the Nan'ao No.1 Shipwreck that sunk between the late 16th to early 17th centuries [63, 74]. Although some scholars have proposed stylistically equivalent ceramics were produced at the Cizao Kilns, Fujian Province, and the Shiwan and Qishi Kilns, Guangdong Province, Wong [63] notes that further provenience research is required. It is likely that each manufacturing centre has is own compositional signature, and data presented in this paper will assist future clarification of South Chinese stoneware storage jar production.

### Discriminating Cambodian brown-glaze (Group D)

Group D is a distinct cluster, most similar to Groups C and B, as demonstrated by the hierarchical cluster analysis (Fig 6). All of the samples within Group D were identified visually as Cambodian brown-glaze stoneware. None of the Sisatchanalai or Bang Rachan reference samples were projected within the ellipse of this cluster. Group D has the lowest coefficient of variation for all elements compared to the other groups (see S3 Table) and, assuming that the compositional recipe was consistent over time, the samples were likely to have originated from the same kiln complex. These sherds are visually similar to Angkorian stoneware ceramics (dark-brown glazes, light-grey paste), but with no diagnostic decorations that can suggest the kiln of origin. We hypothesise this group is from an unrecognised Cambodian brown-glaze stoneware kiln. Are the sherds extraordinary examples of Early Modern Cambodian production, or heirloom ceramics curated from the Angkorian period?

Using the NAA relative standardisation method Grave et al. [18] differentiated four Angkorian kiln complexes dating between the 9th– 13th centuries. The data of NAA relative standardisation and *k*_*0*_ methods can be compared using E_n_ scores, however statistical analysis packages do not produce equivalent results [92]. Nevertheless, there is no relative correspondence between the elemental averages of Angkorian stoneware production [18] and Group D identified in this study (see S3 Table). Although evaluation of elemental averages must be made with caution, the difference between known Angkorian kilns and the Cambodian brown-glaze sherds from Longvek likely relates to an unrecognised production location, and/or temporal differences that characterise Early Modern production.

### Storage jars, Angkorian legacies, and trade

While popular perspectives attribute transformations in Southeast Asian economies to external forces, archaeology can show patterns in material culture that reveal the complexity and nuance of local dynamics [28]. Longvek and Cambodia were connected to the Early Modern period expansion in regional and international trade [8, 35–37]. From a sample of eighty-eight sherds at Longvek, forty-nine sherds were produced in Thailand, and twenty-eight were produced in South China. It is challenging to interpret the rise of mercantilism embodied in trade ceramics against a background of Angkorian decline and contraction of its substantial ceramics industry. Large quantities of trade ceramics, including a few small Chinese brown-glaze jars, are known during Angkorian period but are typically associated with elite use [96–99]. Because of the variability of archaeological contexts and methods, it is difficult to be precise on this subject, but total ceramic imports including porcelain appear to have increased from around or below ten percent at Angkor [97–99] to around fifteen percent at Longvek [10, 46, 47]. From the 15th century consumers at Longvek had mostly replaced locally produced storage jars with imports.

At the same time Longvek looked to foreign trade, fragments of Cambodian brown-glaze stoneware jars verify a connection to the Angkorian past. Compositional analysis NAA identified sixteen sherds of Cambodian brown-glaze storage jars in 15th– 17th century archaeological contexts (Group D). There is a long tradition of storage jar use, manufacture and trade in Cambodian material culture. Radiocarbon ages suggest terminal production dates in the 14th century [100], although some scholars have suggested the kilns of Choeung Ek near Phnom Penh continued to fire after the fragmentation of Angkor [58, 101, 102]. Beavan et al. [21, 22] note the use of Angkorian Buriam or Torp Chey and Bang Rachan brown-glaze jars during the 15th century by upland communities of the Cardamom mountains. Similarly, Angkorian stoneware storage jars continue to be revered by upland communities and ethnic minorities in contemporary Cambodia, Laos and Vietnam [103, 104]. Setting aside the impediments to direct comparison, there appears no correspondence with the Angkorian stoneware elemental groups defined by Grave et al. [18], leading to the proposition that the Longvek Cambodian brown-glaze stoneware sherds are from an unknown kiln. Relatively few fragments and some from contexts that date as early as the 15th century indicate these ceramics likely outlived their Angkorian production date.

At Longvek Thai stoneware sherds are greater in number than Chinese stoneware sherds. These numbers might be a function of the proximity to the kilns, a lingering demand for these specific types of vessels after the demise of the Angkorian ceramic industry, and also changing production capacities at Bang Rachan and Sisatchanalai. Combined with examples of iron under-glaze stoneware [10, 46] Longvek continued to import Thai ceramics notwithstanding verifiable conflicts between the Ayutthayan and Cambodian polities in the second half of the 16th century [36, 38, 42]. Additional investigation is needed, but there may be some correspondence between Chinese brown-glaze ceramic types from 15th century shipwreck sites [11, 63] and Chinese sherds from archaeological contexts at Longvek, opening the way to recognise residual or exceptional trade despite the Ming export ban [11]. Similarly, the administration of trade at Longvek warrants further research, however there is considerable documentation that reveals the innovative mechanisms that facilitating an increase in importation of foreign storage jars. Settlements of traders from China and other polities were established close to Longvek and individuals from different countries held commercial and state positions to facilitate the trade of these goods [36, 105, 106].

## Conclusions

This study presents quantitative multi-elemental analysis of major, minor and trace elements present in ceramics from Longvek, the 16th and 17th capital of Cambodia. Significantly, the application of high-precision NAA has confirmed that elements at 0.1 ppm or below are important discriminatory characteristics to recognise the production origins of Asian stoneware jars [18, 19].

15th– 17th century stoneware jars produced in Thailand and South China found across the globe demonstrate their utility as sturdy containers and exotic objects. By interpreting geochemical data produced by *k*_*0*_-NAA this study offers evidence of forty-nine sherds from Thailand and twenty-eight from South China in archaeological contexts at Longvek and illustrates that Cambodia was part of the Early Modern world. Preliminary examination suggests the ceramic assemblages of Longvek [9, 10] are consistent with finds from shipwrecks and collections across the region [11, 30, 31, 34, 51, 52–54, 56, 57, 63, 78–80].

Although brown-glaze storage jars were recovered from contexts dating no earlier than the 15th century, the majority of diagnostic ceramics linked to stratigraphic contexts from the latter half of 16th century and the first half of the 17th century [9, 10]. This research reveals the predominance of sherds from the Thai kilns of Bang Rachan and Sisatchanalai that may be explained by the proximity of Longvek to Thai production centres. In the wake of a dissolved Angkorian ceramic industry it is not surprising that consumers looked to neighbouring and emerging industrial centres, however we note no evidence of Vietnamese storage jars [62]. Longvek had one foot in the Angkorian era, and sixteen Cambodian brown-glaze sherds identified by *k*_*0*_-NAA are likely vessels used into the Early Modern period produced by an unknown and obsolete Angkorian kiln. Both familiar and new forms of material culture embodied in brown-glaze storage jars identified by *k*_*0*_-NAA demonstrate that Cambodia was engaging with new forms of Early Modern mercantilism and after Angkor opening up to Southeast Asian modernity.

## Supporting information

S1 Data Sample *k_0_*-NAA raw data. (XLSX)

S1 Table Sample list. (XLSX)

S2 Table Summary of the first 10 principal components with variation and cumulative percentages. (XLSX)

S3 Table Summary NAA results for groups by average (Av.) and coefficient of variation (C.V). Reported as ppm.
(XLSX)
